# Recent Progress in the Preparation Technologies for Micro Metal Coils

**DOI:** 10.3390/mi13060872

**Published:** 2022-05-31

**Authors:** Jianyong Lou, Haixia Ren, Xia Chao, Kesong Chen, Haodong Bai, Zhengyue Wang

**Affiliations:** State Key Laboratory of Electrical Insulation and Power Equipment, School of Electrical Engineering, Xi’an Jiaotong University, Xi’an 710049, China; hxren@stu.xjtu.edu.cn (H.R.); chaoxia@stu.xjtu.edu.cn (X.C.); 3121104019@stu.xjtu.edu.cn (K.C.); baihaodong@stu.xjtu.edu.cn (H.B.); 3120304033@stu.xjtu.edu.cn (Z.W.)

**Keywords:** micro metal coil, MEMS processing technology, flexible electronic process, magnetic film, printing process

## Abstract

The recent development of micro-fabrication technologies has provided new methods for researchers to design and fabricate micro metal coils, which will allow the coils to be smaller, lighter, and have higher performance than traditional coils. As functional components of electromagnetic equipment, micro metal coils are widely used in micro-transformers, solenoid valves, relays, electromagnetic energy collection systems, and flexible wearable devices. Due to the high integration of components and the requirements of miniaturization, the preparation of micro metal coils has received increasing levels of attention. This paper discusses the typical structural types of micro metal coils, which are mainly divided into planar coils and three-dimensional coils, and the characteristics of the different structures of coils. The specific preparation materials are also summarized, which provides a reference for the preparation process of micro metal coils, including the macro-fabrication method, MEMS (Micro-Electro-Mechanical System) processing technology, the printing process, and other manufacturing technologies. Finally, perspectives on the remaining challenges and open opportunities are provided to help with future research, the development of the Internet of Things (IoTs), and engineering applications.

## 1. Introduction

Over the last few decades, the field of micro metal coils has evolved rapidly due to the widespread application of micro metal coils in fields such as consumer electronics, the automobile industry, aerospace, machinery, the chemical industry, medicine, among others [1,2,3,4,5,6,7,8,9,10,11,12,13,14,15]. Among the various types of micro metal coils, the coils based on MEMS technology and flexible electronic technology have greater research value [1,2,3,4,5,6,7,8].

The coil turns of micro metal coils are greatly increased in a limited volume, resulting in a more uniform, high-density magnetic field when working [9]. They have the advantages of miniaturization, integration, intelligence, low cost, high performance, and mass production, which are prerequisites for realizing a high level of integration and complexity in modern electronic equipment [12,13,14,15,16]. The micro-transformer produced by micro coil technology has low resistance, high inductance, a high coupling coefficient and quality factor, and plays an important role in switching the power supply, power conversion or signal isolation [15,16]. In particular, the DC–DC converter that is composed of a micro-transformer effectively reduces the volume of the power system and is widely used in various portable electronic products. The application of micro coils in relays makes the size of the aeroengine shrink, which promotes the development of micro-aviation [17,18,19].

Compared with the traditional metal coil, the flexibility and ductility of the flexible coil are significantly improved, and it is easy to bend and fold, as well as being lightweight [5,6]. As the most efficient energy supply mode in micro-energy acquisition technology, an electromagnetic energy harvester also uses flexible coils to solve the problem of ensuring high output voltage under the premise of controlling the volume [20,21,22]. Micro coils can be nested on wearable system devices. Flexible sensors convert external deformation signals into electrical signals. MEMS sensors can achieve almost all sensory functions of the human body, including vision, hearing, taste, smell (such as the Honeywell electronic nose) and touch, which play an important role in health monitoring, motion monitoring, swallowing speech monitoring and intelligent rehabilitation [23,24,25,26,27,28,29]. With the development of micro coils, people can directly obtain certain body information in a convenient, fast, and friendly way. The wearable device based on micro coil technology is advantageous due to its small size and portability. It deeply contacts the measurement target, reduces the cost, and completes the integration of multiple functions with a certain level of accuracy [26,27,28,29,30,31,32,33]; therefore, the application of micro metal coils has greatly expanded the use of electronic devices and laid the foundation for the rapid development of consumer electronics, the automobile industry, and even the aerospace, machinery, chemical, and medical monitoring industries.

The development of micro metal coils also benefits from the significant progress of advanced microfabrication technologies, such as microelectronic technology, integrated circuit technology, and its processing technology, MEMS preparation technology, and film and flexible electronic processing technology [34,35,36]. The substrate materials mainly include silicon, glass, ceramic materials and flexible materials [37,38,39,40,41,42]. The hard substrate has a high degree of hardness and good dielectric properties, but for some non-planar surfaces and curved surfaces, it is necessary to use a flexible substrate to prepare sensors. At present, the organic polymer materials for flexible substrates studied by scholars in China and abroad not only have better mechanical properties, dielectric properties, and physical and chemical stability, but also have good mapping ability and flatness, and are widely used in the manufacturing of MEMS sensors [39].

Micro metal coils are mainly divided into two structural types: three-dimensional coils and planar coils. Three-dimensional coils are mainly solenoid-type micro coils, and planar coils can be subdivided into single-layer coils and multi-layer coils [40,41,42]. The single-layer planar coil has no magnetic core-binding circuit, and the magnetic leakage is significant. Then, the single-layer thin film magnetic core or the two-layer thin film structure and sandwich structure are introduced to remarkably improve the inductance and quality factor of the planar spiral inductor [2,3,4,5,43,44,45,46]. The solenoid based on the MEMS process has good integration performance on standard silicon substrate, and the hollow solenoid coil can reduce the influence of substrate [47,48,49,50]. Three-dimensional solenoid inductors and suspended planar inductors were fabricated on silicon and glass substrates using thick gel lithography and copper plating. Moreover, a high-performance, folded, stacked multilayer 3D magneto-inductor coil was fabricated using MEMS technology. The new folding and welding methods successfully solved the complexity of the key manufacturing process of multilayer inductors, enabling the simple, rapid and low-cost assembly of multilayer coils [47,51,52,53,54,55].

In this paper, the structure, preparation methods and preparation materials of micro coils are summarized. Section 2 discusses the characteristics and applicable scope of coils with different structures. The study of micro metal coils based on different processing methods and materials is emphasized in Section 3. Finally, we provide typical devices and applications of coils based on different processing methods. The challenges and open opportunities of the micro metal coil preparation process for future development are given.

## 2. Type and Theory of Micro Metal Coil Construction

### 2.1. Theory of Coil Performance

When conducting theoretical analyses of a coil, the inductance, quality factor Q and distributed capacitance of the coil are generally of highest concern. The degree of inductance of the coil depends mainly on the number of turns in the coil, the winding method, the presence or absence of a magnetic core, and the material of the core. In general, the more coils there are and the denser the wound coils, the greater the inductance. Coils with a center of gravity have a larger inductance than coils without a center: the larger the permeability of the core, the greater the inductance. The higher the quality factor Q of a coil, the smaller the loss and the higher the efficiency. The Q value of a coil is related to the DC resistance of the wire, the dielectric loss of the skeleton, losses caused by the shield or iron core and the influence of high-frequency skin effects. There are certain capacitances between the turns, between the layers of any inductance coil, between the coil and the reference ground, and between the coil and the magnetic shield. These capacitances are called the distributed capacitances of inductance coil. If these distributed capacitances are integrated, they will become an equivalent capacitance, C, in parallel with the inductor coil. The existence of distributed capacitance decreases the Q value of a coil, and the stability thus deteriorates; therefore, the smaller the distributed capacitance of the coil, the better.

**Figure 1:**
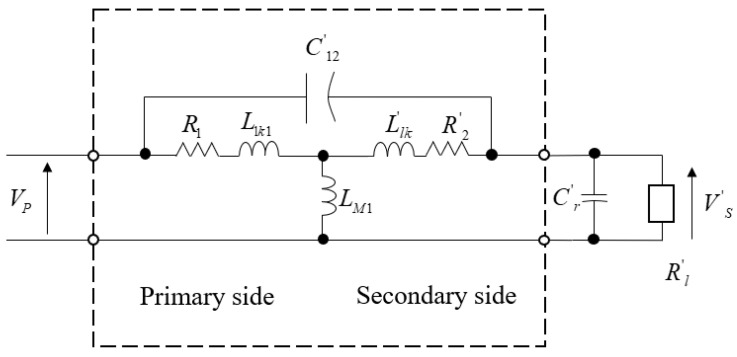
Circuit model of transformer.

Due to the complex configuration and diversity of coils in practical applications, it is difficult to carry out complete theoretical calculations; as a result, the simplified model and equivalent circuit are hard to explain. Below, a PCB board coil is presented as an example [56]; qualitative analysis was used to determine the performance of the coil transformer. The transformer circuit model is shown in Figure 1.

The inductance was measured using an HP4194A impedance/gain phase analyzer. The stray inductance of primary winding could be compensated by the impedance analyzer; however, lead stray inductance between the secondary winding and the external capacitor could not be compensated as it must be included in the transformer model as part of the secondary leakage inductance. The capacitance inside a winding is far smaller than the external capacitance; therefore, their influence was ignored to simplify the analysis.

The voltage gain ratio and no-load resonant frequency of the transformer could be determined by connecting an external capacitor to the secondary winding and the measured coil inductance. This frequency characteristic is an important factor in the design of power converters for a specific switching frequency range.

### 2.2. The Solenoid

**Figure 2:**
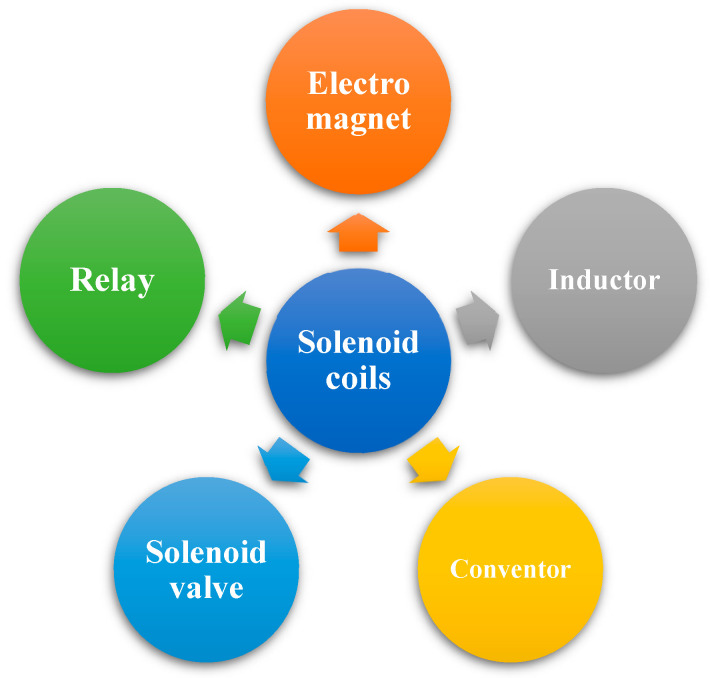
Main applications of solenoid coils as common electromagnetic components.

The solenoid coil is a three-dimensional coil in space, directly wound by metal wires. Its interior is either hollow or a metal core, and it is used to concentrate the magnetic field. When passing a current in a closed loop, the magnetic field can be generated. As a common electromagnetic component, the solenoid is widely used in various fields, as shown in Figure 2; it is used for electromagnets [57], inductors [58], converters [59], solenoid valves [60], relays [61], and so on, as a part of inductors and transformers.

Moazenzadeh et al. [11] formed a vertical magnetic core column using a laminating process. The magnetic core material is a cobalt-based amorphous magnetic alloy, and the column is arranged on a 4-inch borosilicate silicon sheet with adhesive. The column is divided into a T column and a U column. On the T column, the solenoid is directly wound on the magnetic core by the lead-bonding machine, and then combined with the U-shaped magnetic core to form a closed magnetic circuit. The wiring bonding process is fast, and up to 40 coils can be made in 10 s. The manufactured device generates an inductance of 2.95 μH/mm^3^ and 133 nJ/mm^3^ in energy per unit volume at a frequency of 1 MHz. Coupling coefficients of up to 98% and power efficiencies of 64–76% for different turn ratios have been measured.

Hao et al. designed transformers with magnetic thin films for on-chip power conversion and isolation [13]. The transformer is composed of three metal layers separated by insulators, including the bottom copper layer, the top copper layer, and the middle core layer. A cobalt alloy thin film material with high permeability is used as the magnetic core, the coil is wound on the magnetic core, and the coil axis and core are parallel to the silicon substrate. It is manufactured by using standard CMOS (Complementary Metal Oxide Semiconductor) manufacturing equipment and processes. By using a high-permeability, magnetic material as the solenoid core, the inductance of the device is greatly improved, with an inductance density of up to 108 nH/mm^2^. The transformer’s construction has a total thickness of less than 30 µm and a volume inductance density of 3.6 µH/mm^3^. By using the lamination core, the loss is controlled, and the peak quality factor (Q) at 40 MHz is 16.

The significant advantage of the solenoidal is that the spatial structure is regular, the magnetic field formed is evenly distributed in space, and the electromagnetic conversion loss is low when the same materials are used. Moreover, magnetic conductive materials can be placed in the micro-channel, which is surrounded by metal wires, to further reduce losses and constrain the magnetic field, as well as to improve the capacity and the signal’s sensitivity. The closed flux chain is formed by the closed magnetic core; thus, the magnetic leakage can be well prevented. The solenoid coil can also achieve a higher inductance per unit area, and it is more convenient to increase the inductance value by increasing the coil’s cross-sectional area.

### 2.3. Planar Micro Metal Coil

The biggest advantage of planar micro coils over solenoid-type micro coils is that mass production is cheaper and more efficient when the required size is smaller. The plane-type micro coil is different from the solenoid-type micro coil in structure and has a planar two-dimensional structure. Planar micro coils were first used in MRI (Magnetic Resonance Imaging) as surface coils [62]. The structure of the planar micro coil usually includes the upper and lower magnetic films, a metal wire coil layer in the middle, and an insulating layer used to isolate the magnetic core material and the wire.

The main structural parameters include the outer diameter of the micro coil, winding number, line width, coil spacing, and thickness. When multiple layers of coils are stacked, the number of layers also needs to be considered. Wu et al. embedded two interlaced thick copper coils in the bottom layer of silicon substrate, and prepared transformer coils with a power IC process combined with MEMS electroplating, photolithography, and deposition. Through-holes and coil grooves are etched on the surface of silicon wafers by photolithography, and low-temperature oxide deposition is followed by electroplating copper filling [16]. Additionally, a new kind of silicon-embedded coreless transformer (SECT) for isolated DC–DC converter applications is presented and demonstrated. By embedding two staggered, thick copper coils in the bottom layer of the Si substrate, the designed 2 mm^2^ SECT can achieve a maximum transformer efficiency of 85% at 50 MHz. Compared to previously reported silicon-based coreless power transformers, the lower operating frequency of SECT allows for about a 50% reduction in power losses in the power MOSFETs and Schottky diodes, resulting in a 38% reduction in converter losses.

Wang et al. designed a silicon-integrated micro-transformer for an isolated bias power supply [63] and carried out fabrication and characteristic verification. The racetrack-shaped micro-transformers are designed and manufactured using an advanced double-layer metal (DLM) micromachining process. The inductance density of the DLM device exceeds 80 nH/mm^2^ and the efficiency is about 78.2% at 20 MHz with a 0.5 W output. The inductance drop is less than 20%, the bias current is 0.35 A, and the DC breakdown voltage can reach 6 kV. The racetrack-type micro-transformer is designed and manufactured by a double-layer metal (DLM) micromachining process. The bottom magnetic core is electroplated on a natural oxide insulating silicon wafer to form a pattern, and two layers of copper windings are deposited on the BCB and the SU–8, with the SU–8 as the middle insulating layer. From bottom to top, there are the bottom core, the first insulating layer, the first copper coil, the second insulating layer, the second copper coil, the third insulating layer, and the top core.

Silicon-based substrates have the advantage of forming metals directly, compared with flexible substrates. By using the planar coil, we can integrate the conductor on the plane, which improves the utilization rate of axial space and increases the cross-sectional area of the magnetic core. The manufacturing process is simpler, and it is more widely used when the demand for miniaturization is higher [64]. Moreover, a higher power density can be achieved [65].

When the number of turns of the metal coil needs to be increased, and when the required number of turns exceeds a certain level, the plane area occupied by the planar coil will increase rapidly, which is not conducive to the reduction in the system’s size and application in a narrow space.

### 2.4. Integrated Micro Coil with Flexible Substrates

Micro coils based on flexible substrates have the characteristics of bendability, biocompatibility, being lightweight, and having a low base cost. Due to the poor flexibility and ductility of the rigid base, the electronic components used in the conditions that need to be deformed cannot be on the rigid base, and they must be put on flexible substrates. Flexible electronics have broad application prospects in many fields such as healthcare, environmental monitoring, display and human–computer interaction, energy conversion, management and storage, communication, and wireless network technologies [25].

Yang et al. [66,67] adopted different MEMS processing techniques for different core materials in their research, including an electroplated nickel–iron alloy iron core and a pasted cobalt-based amorphous strip iron core. The preparation of copper coils is achieved via processes such as oxidation, photolithography, sputtering, electroplating, and etching. After the processing on the hard material is completed, the processing of the flexible substrate coil device is completed by peeling, planarizing, and pasting on the flexible substrate. The flexible base fluxgate adopts a parallel excitation method, and the primary and secondary coils are alternately wound with each other. The coil line width is 180 μm, the gap is 120 μm, the total number of turns of the coil is 100, the turn ratio of the excitation coil and the induction coil is 1:1, and the thickness of the copper wire is 4μm. The coil’s spirally wound iron core is a long strip iron core with a length of 30 mm and a width of 3 mm. The thickness of the electroplated nickel–iron alloy iron core is 4 μm, the thickness of the pasted drill-based amorphous strip iron core is about 5 μm, and the pad size is 1350 μm × 1350 μm.

The flexible substrate miniature metal coil makes the device better for miniaturization and integration, and due to the large degree of deformation, the application range is wider than that of the rigid substrate. Moreover, the coil stacking is more convenient, which is convenient for improving the power density of the device. Since some parts of the metal coil need to be processed in a high-temperature environment, processing directly on the flexible film will cause damage to it. It needs to be prepared on a rigid substrate and then transferred to the flexible film, which increases the complexity of the processing technology. Moreover, since only soft magnetic material can be used as the iron core on the flexible substrate, the electromagnetic properties are weaker than traditional materials, and further research on the required soft magnetic material is required.

## 3. Main Processing Method

### 3.1. Macro Production Mode

#### 3.1.1. Processing on PCB

The planar micro coil based on the printed circuit board (PCB) process is advantageous in terms of having a low manufacturing cost, short cycle time, and the ability for mass production.

Wei P.W. et al. [68] designed a PCB-based planar micro coil for portable NMR (Nuclear Magnetic Resonance) detection. The PCB plane micro coil is made on the double-sided PCB using a photolithography and etching process, the micro coil is obtained after the copper wire is deposited by exposure, development, and electroplating, and the two sides of the PCB are electrically connected through the PCB through-holes. The top board includes the left and right pads, the spiral micro coil and the leads. The leads are used for the connection between the left pad and the spiral micro coil. There are two through-holes in the middle of the PCB board, and the innermost end of the spiral coil and the pad on the right side of the top board are connected to the bottom board with wires through the two through-holes. Wei P.W. et al. connected the two through holes with leads on the bottom plate, so as to realize the connection between the spiral coil and the pad. In order to avoid the problems of the thin glass’ intermediate layer being easily broken and the flat micro coil being easily oxidized, it is necessary to improve the structural design and adjust the subsequent process’ production plan.

Although the current PCB planar micro coils are relatively inexpensive to manufacture, they have shortcomings, such as numerous steps in the process, low manufacturing resolution, and uneven RF (Radio Frequency) fields. It was found that the parasitic capacitance caused too much influence, due to multiple through-holes, to make a PCB three-dimensional coil in the study. In order to obtain a high signal-to-noise ratio (SNR), PCB planar micro coils need to be properly designed. The width of the wires and the distance between wires should be as small as possible. Due to the limited minimum resolution of PCB technology, the wire’s width and spacing are both 152.4 μm, the coil’s thickness is 35 μm, and the number of turns is six. Tang et al. [56] designed an isolated switching power converter coil for a coreless PCB transformer. The transformer coils are micro two-spiral windings, which are printed directly on both sides of a double-sided PCB. The PCB laminate is made of FR4 material with a high breakdown voltage.

Previous micro-transformers typically used ferrite cores or materials to provide a closed magnetic circuit; however, the rated current and operating frequency are limited by the ferrite material due to magnetic saturation and eddy current losses. By printing the windings of the planar transformer on both sides of a double-sided printed circuit board (PCB), manual winding costs are eliminated, and the manufacturing process is facilitated. As the transformer windings are etched on the PCB surface, encapsulation processes and materials such as epoxy can be excluded. In addition to the device package, the magnetic core is eliminated, so the height of the device can be greatly reduced.

Coreless PCB transformers eliminate the application challenges of core-related transformers in low-power scenarios. The diameter of the coreless PCB transformer is about 0.46 cm, the power output of the converter is about 0.5 W, and the typical transformer efficiency is 63%. Its excellent high-frequency capability, high reliability, and low-profile construction make coreless PCB transformers a suitable choice for switching converters and microcircuits in the megahertz range.

#### 3.1.2. Wire-Wound Coils

(1) Hand winding

Based on the capillary’s characteristic of not only being able to store samples but also being able to serve as a coil bobbin, Peck et al. [69] made 28 solenoid micro coils with diameters of 50 μm to 1.8 mm by manually winding wires on the capillary. The experiment for detecting the electrical parameters of coils and the NMR experiment both show that the fabrication method is feasible, and the experiment also verifies that reducing the diameter of the solenoid micro coil results in an improvement in the detection sensitivity. In the same research group, Olson et al. improved the method of fixing the solenoid micro coil and replaced the epoxy resin with cyanoacrylate adhesive. In the experiment, the cyanoacrylate adhesive was able to penetrate into the gap between the capillary and the wire more easily. At the same time, the FC–43 solution was used to immerse the coil to reduce the magnetic susceptibility mismatch between the coil and the sample. The experimental results showed that the detection sensitivity was improved by one to two orders of magnitude. The advantage of the manual winding method is that the method is simple and suitable for manual operation; the disadvantage is that it is not easy to manufacture in batches.

(2) Wire Bonding

Based on the MEMS process, Kratt et al. [70,71] used wire bonding technology to wrap the solenoid coil wire on SU–8. First, on a silicon or glass substrate, they sprayed a layer of chrome/gold (50/500 nm) and an AZ 1518 photoresist with a UV (Ultraviolet) pattern, and wet etched the chrome/gold layer to reveal the metal that will facilitate the connection of the wire-tail’s pad. Next, SU–8 pillars were fabricated on a 4-inch wafer by photolithography. Different from the traditional experience of spin-coating photoresist, the research team of the author’s research group first calculated the SU–8 based on the thickness of the photolithography target. A spin-coating volume of 8 was required for the photoresist. Finally, the wire (gold wire) of the solenoid micro coil was wound in four steps: the first step was wire bonding; then, the metal pad and the gold wire ball end were treated with plasma; next, the ball end was ultrasonically welded; and then, the wire was helically wound. Finally, the end of the wire was ultrasonically re-soldered. The wire-bonding winding method is characterized by being a novel method and having the potential for mass production. This method’s disadvantages include the fact that the coil fabrication is subject to a lithography process, as well as the mechanical strength of the pillars. Table 1 shows a summary of the examples of macro production mode.

### 3.2. MEMS Processing Technology

It is difficult to fabricate miniaturized and high-performance multilayer micro coils on planar substrates with conventional microelectronic technology. In recent years, the rapid development of micro-electro-mechanical systems (MEMS) technology makes it the most advanced technology in the development of miniaturized multilayer structure micro-inductors and RF MEMS devices in the world [72]. This technology is characterized by its miniaturization, diversity, and compatibility with microelectronic technology, and its processing size is generally between 1–100 μm. Products based on MEMS technology have the advantages of small size, easy integration, high sensitivity, low power consumption, high reliability, and being lightweight. The micro coil can simply be divided into a single-layer planar coil, solenoid coil, and multi-layer planar coil from the structure. The MEMS processing technology of these three types of coils is briefly introduced below.

#### 3.2.1. Three Types of Micro Metal Coils Based on MEMS Technology

(1) Single-layer planar coil

With the rapid development of thin-film technology and microelectronics technology, magnetic thin film inductance devices are also developing toward miniaturization and integration. The most representative magnetic thin film inductance devices were proposed by Soohoo [73] in 1979 and developed in 1984 by Kawabe et al. [74]. They studied various planar micro-inductors, including single-turn, winding-type, spiral-type, zigzag-type, micro-inductors, and so on. Afterwards, the research on magnetic thin film micro-inductors began to advance very rapidly. Compared with other types of inductors, planar spiral inductors are simple to manufacture, they have good compatibility with the IC process, and they also have a low production cost. Planar spiral inductors also have the advantages of a wide frequency range and high performance at a high frequency; however, they also have small inductance at a low frequency, which is usually several NH, and they also experience resistance loss, eddy current loss, parasitic capacitor loss, and other defects. As there is no magnetic core-binding magnetic circuit, serious magnetic leakage can occur, which will interfere with the vertically integrated device [45].

**Figure 3:**
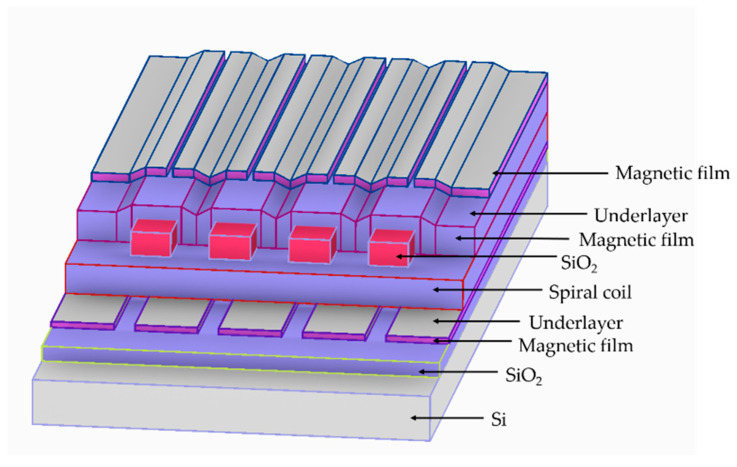
Schematic diagram of a sandwich inductor with a magnetic thin film.

**Figure 4:**
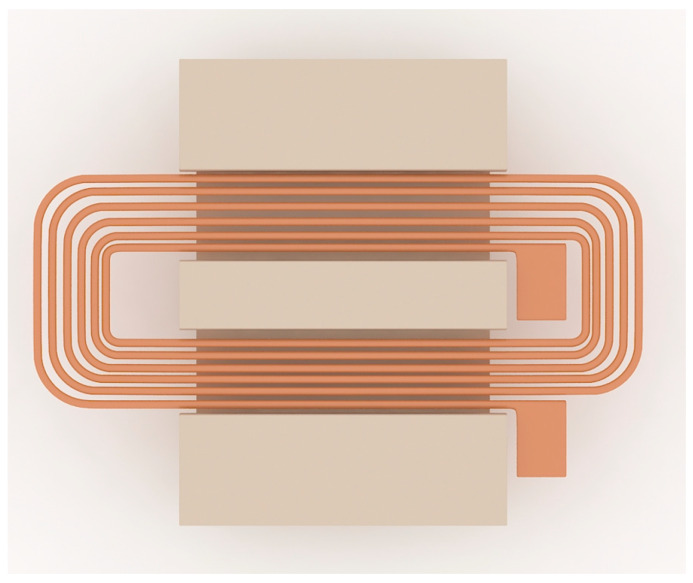
Sandwich structure with electroplated NiFe as the magnetic core.

To solve the magnetic flux leakage problem of planar spiral inductors, monolayer film is introduced in the hollow planar spiral inductors and magnetic core, and is distributed in the planar coil of the upper or lower section. Alternatively, two layers of magnetic core are used, and the spiral coil is placed between the two magnetic films and separated by insulating material between the coil and the magnetic thin films. The use of soft magnetic materials with high permeability at high frequencies will significantly improve the inductance and quality factor of planar spiral inductors. For example, Yamaguchi et al. [46] from Japan reported in 2000 that RF-integrated magnetic thin film micro-inductors were sputtered on the spiral coils. A layer of resistive oxide magnetic film is added at the bottom of each layer to form a sandwich structure (Figure 3). Its DC resistance is 6.8 Ω. Compared with the single-layer magnetic film structure, it is more conducive to anti-magnetic leakage, and the inductance performance is improved at 2 GHz (L = 7.9 nH, Q = 12.7). Compared with the same type of inductance with an air core, the performance is improved by 19% and 23%, respectively. The inductance is 7.7 nH at 1 GHz and the quality factor is 7, which is a key step towards the application of RF–MEMS for magnetic thin film micro-inductors. Peng S [75] also used a similar sandwich structure to build a micro-inductor on a silicon substrate. The micro-inductor has a single layer of copper winding sandwiched between two layers of electroplated NiFe core (Figure 4). It has a footprint area of 2.9 mm^2^, an inductance of 204 nH at 21.7 MHz, a DC resistance of 470 mΩ, and a peak quality factor of 9.23 at 9.2 MHz.

In the late 1980s, portable electronic products including mobile communication products such as mobile phones, notebook computers, and computer microprocessors created higher requirements for miniaturized DC–DC converters composed of magnetic thin film micro-inductors and transformers. There were also higher requirements for frequency and power conversion efficiency for switching the power supply; therefore, increasing the switching frequency and reducing loss have become inevitable trends. The operating frequency of the magnetic thin-film micro inductor is increased from 1 KHz–1 MHz to 1–10 MHz, the inductance is greater than 1 µH, the quality factor is greater than 1, and the conversion efficiency is greater than 95%.

**Figure 5:**
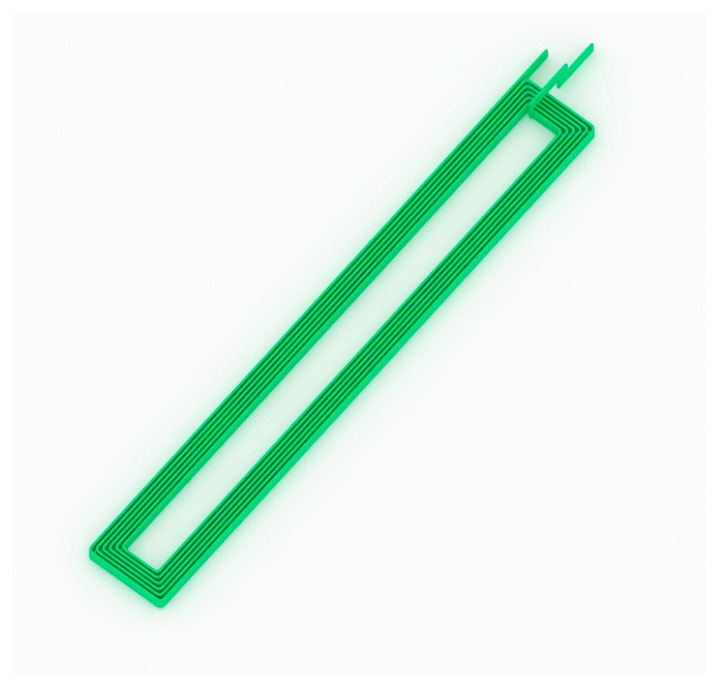
The inductors, with a size of 14.88 × 2 mm^2^, are simulated onto a substrate of 525 μm of silicon +1 μm of silicon dioxide.

To improve the quality factor and self-resonant frequency of the inductor, researchers have conducted a lot of research. In 1999, K. Kamogawa et al. developed a MEMS planar helical inductor [76]. The inductance structure was developed on a 10-μm polyimide thick film insulation layer, a 0.7-μm-thick grounding aluminum layer was buried between the polyimide layer and the substrate 45.77, and the self-resonant frequency was 29.3 GHz. The maximum Q value was 45.77. In Switzerland in 2014, Jacopo Olivo et al. [77] created an inductor coil that can be used in implantable biosensors (Figure 5). The inductor is 14.88 mm × 2 mm in size and is backed by 525 μm of silicon with a 1-um silicon dioxide layer. The inductance coil is made of copper material with a thickness of about 60 μm. As the thickness of the inductance coil is increased, the quality factor of inductance will be improved. The self-resonant frequency is located at 31.3 MHz. At 5 MHz, the inductance value is 0.46 μH, and the Q factor is 13.65.

(2) Multi-layer planar coil

**Figure 6:**
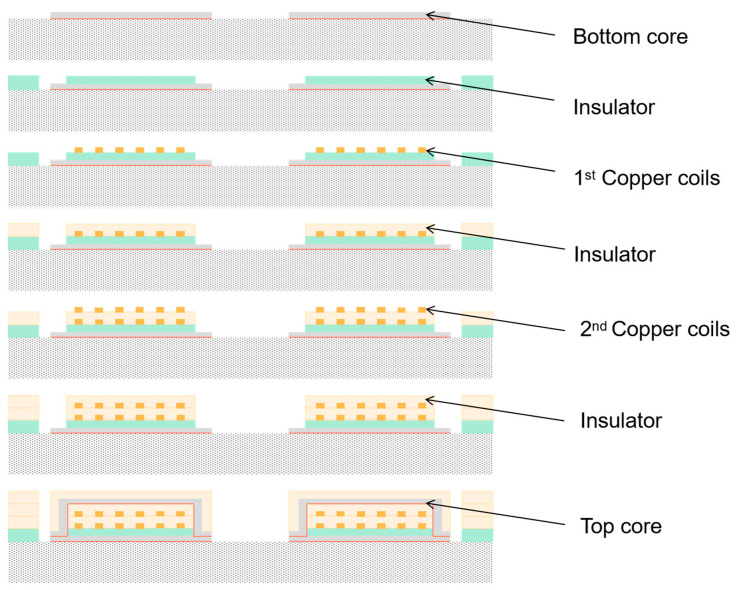
Double coil preparation process.

With the progress of technology and the reduction in the device’s feature size, the area consumption caused by planar inductors has become increasingly obvious. A great deal of research has been carried out on the fabrication of miniaturized on-chip inductors with a high-quality factor and a high resonant frequency. Lakdawala et al. [78] studied the suspension inductance realized by the reactive ion etching (RIE) process; however, this method does not reduce the occupied area of the inductor. Yoon et al. [49] fabricated a three-dimensional solenoid inductor and a suspended plane inductor using the multiple exposure single development (MESD) method on silicon and glass substrates using thick gel lithography and copper electroplating. Chen et al. [79] also achieved a similar structure with SU_8 glue. This method can effectively improve the Q value of inductance and can reduce the inductance area. Three-dimensional coils, especially stacked multilayer inductors, are another option that can utilize vertical space to save on area and improve performance [80,81,82]. For example, in [81], the inductor can achieve an L of 60 nH and a Q of 17.5 at 70 MHz by using a 3D torus design. According to [82], for a multi-layer stacked structure, increasing the number of layers can significantly improve Q and L. Figure 6 shows the preparation process of a double-layer coil.

In 2014, Sun et al. produced a high-performance folded multilayer stacked 3D magneto-inductance coil using the MEMS process [51]. The coil has six layers of Cu material, a soft Ni_80_Fe_20_ magnetic core as the coil center, and soft parylene insulating material as the substrate, and can be used for wireless power transmission. This coil uses a novel folding and welding method, which successfully solves the complexity of the key manufacturing process of multilayer inductors and enables multilayer coils to assemble and fold the area of a six-layer inductor coil simply, quickly and at a low cost. When the inductance density is 100 nil/RAM2 at a 4.1 MHz operating frequency, the inductance value increases from 0.4279 μH to 12.79 μH with the increase in the layers from L to 6, with an increase of 30 times, and the Q value increases from 7.48 to 10.68, with an increase of 43%.

(3) Solenoid Coil

The majority of the first MEMS research was on the study and applications of inductors with the planar coil type because this process is relatively simple; however, even with the constant improvement in terms of technological progress and requests for the planar coil type, there are still many deficiencies. In many cases, it cannot even be used, and the MEMS-type solenoid inductors with small chip areas experience small eddy current loss, and magnetic flux can occur. It is attracting more attention because it increases efficiency by focusing on areas where the driving force is needed.

**Figure 7:**
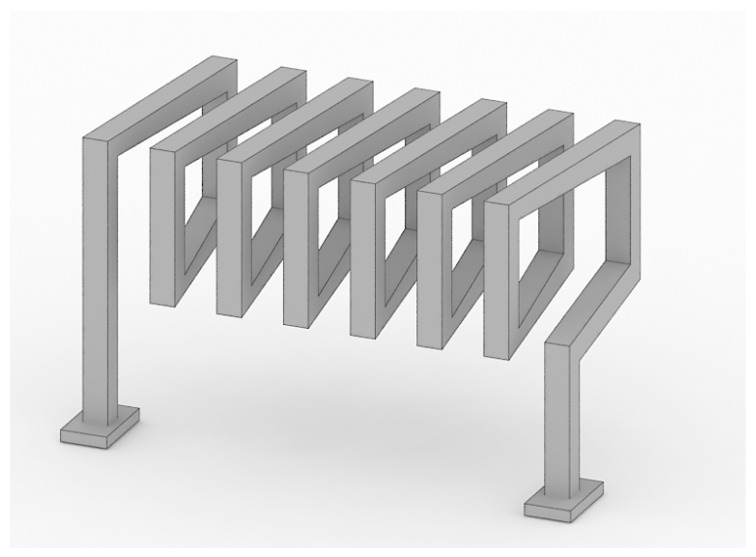
A schematic of an integrated solenoid-type inductor with an air gap.

**Figure 8:**
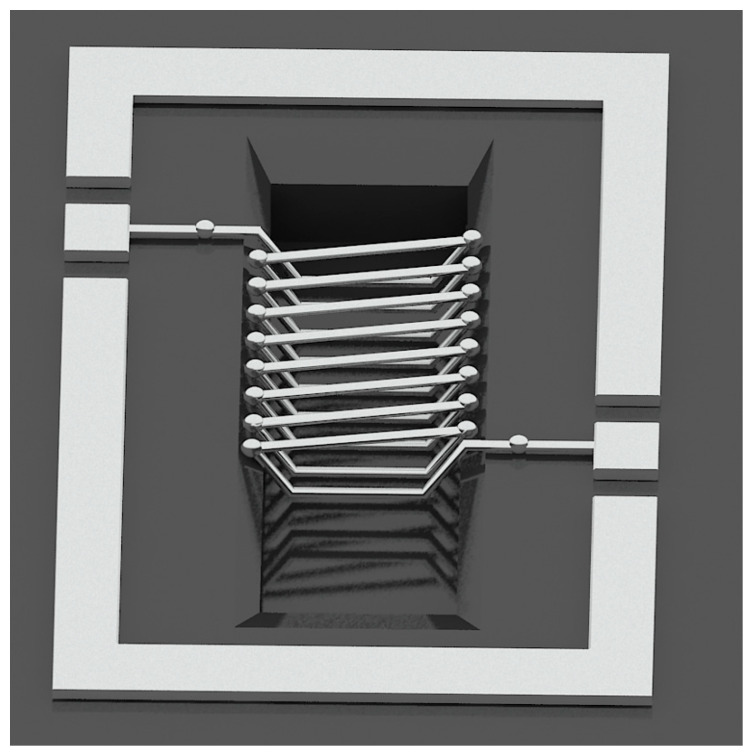
Structure of concave-suspended solenoid inductors.

Dragan et al. [48] fabricated micro-inductors using soft magnetic CZT (Co–Zr–Ta) material as the core material. Its DC resistance is 320 mΩ, and its inductance values are 100 nH at 15 MHz and 97 nH at 30 MHz. The peak Q factor of about 15 occurs at a frequency of 30 MHz. Y.J. Kim et al. [49] proposed an inductor with a hollow ring-type geometry that introduces the air gap between the substrate and the conductor (Figure 7), which reduces stray capacitance, achieving a high Q value as a result. In addition, they studied the various effects of geometric factors. Various inductors with the inductance varying from 1 to 20 nH, and a maximum Q factor from 7 to 60, have been fabricated and measured. Lei Gu et al. [83] etched the silicon substrate into a sunken cavity, embedded the solenoid inductance in the cavity, and suspended it in the cavity, as shown in Figure 8. The loss of substrate is effectively reduced, the mechanical strength is very high, and the influence of environmental vibration is very small. Its DC resistance is 1.27 Ω, and at 5.35 GHz, the maximum value of inductance is up to 45 nH. Similar structures have been reported in the literature [50]. Table 2 summarizes the different structures of metal coils based on MEMS machining technology.

#### 3.2.2. Materials and Processing Technology

From the point of view of MEMS processing technology, the substrate material will be different according to the use of the micro coil, and due to some special application scenarios, such as the requirements of the aerospace environment, insulation performance is also impacted. The following three parts will introduce the base material, insulation material, and coil deposition process.

(1) Substrate material

MEMS process coils can be fabricated on rigid substrates depending on the application. For example, the material properties of silicon are very suitable for the substrate material, which will be crucial for the rapid development of silicon micromachining technology in the future. Glass materials have high light transmission, high hardness, corrosion resistance, excellent biocompatibility, and other material properties, and are often processed into micro-reactors, micro-pumps, micro-accelerators and other devices in MEMS [37]. Ceramic materials, especially engineering ceramic materials, have high strength, corrosion resistance, high temperature stability, and excellent mechanical properties, and are often used to process microturbines, micro tools, and micro substrate materials [38]. The application of insulating hard and brittle materials, such as glass and ceramics, has shown a good momentum of development, and new sensors made of hard and brittle insulating materials, such as MEMS and CMOS, are increasing in demand in the Internet of Things, smartphones, and other industries. For example, sapphire glass is selected as the raw material for the fingerprint identification sensor, camera, and even display panel of Apple mobile phones. This material has extremely high hardness and good dielectric properties, showing strong performance [39].

Although these rigid substrates have a variety of applications, the study of flexible substrates is particularly important for some non-planar and flexible surface measurements, such as the measurement of tubular magnetic field distribution, wearable sensors for the measurement of human or biological surface magnetic fields, and magnetic field sensors for stress measurement. At present, the organic polymer materials used for flexible substrates are PI(polyimide), PDMS(Polydimethylsiloxane), parylene, polyethylene terephthalate (PET), silicone resin, and so on. Compared with traditional Si-based hard substrate materials, organic flexible substrate materials, in addition to being flexible and bending, also have good mechanical properties, dielectric properties, good physical and chemical stability, and so on. At the same time, these materials also have a good drawing ability and flatness, making them widely used in the manufacture of MEMS sensors. Table 3 summarizes the applications of different substrate materials.

(2) Insulation materials

When multi-layer coils are involved in the preparation of coils, an insulating layer is needed to separate the two coils. Common insulating materials are SiO_2_, polyimide, AL_2_O_3_, BN, MgO, and so on. Polyimide can be used as a dielectric layer insulation material between the conductor of the inductance coil and between the conductor and magnetic core. Under the condition that the dielectric constant is similar to SiO_2_, it can realize a thicker insulation layer than the PECVD (Plasma-Enhanced Chemical Vapor Deposition) SiO_2_ layer (thickness is generally less than 1 μm), which can reduce various capacitance values in the device to improve the purpose of the inductance’s Q value. Al_2_O_3_ has a high dielectric constant (about 8.1), very low metal ion permeability, strong radiation resistance, good chemical stability, high thermal conductivity, and other characteristics. Moreover, its insulation is very good, and its resistivity is 3 × 1015 Ω·m. The resistance of most insulating materials decreases exponentially with an increasing temperature at high temperatures. In this case, it is not advisable to increase the resistance simply by increasing the thickness of the insulation layer, not only because the deposition rate of the insulation layer is slow, but also because the increase in the thickness of the insulation layer also indicates an increase in the residual stress, which will lead to the insulation layer falling off.

Researchers have also performed a lot of research on the temperature resistance of the insulating layer. Hiroshi Nakai et al. [85] showed that the insulation layer prepared under the conditions of an ion beam sputtering angle of 40–45, ion beam energy of 10 keV, and substrate temperature of 800 °C has the best insulation performance at the high temperature of 800 °C. The insulation resistance of the 6.5 μm alumina layer at 820 °C is greater than 10 MΩ. The Department of Chemical Process Metrology in the United States [86] focused on the influence of transition layer materials and gas composition in the process of thermal oxidation on the microstructure, composition, and insulation of the thermal oxide layer. The insulation resistance of the thermal oxidation layer and the sputtering alumina composite insulation layer on the NiCoCrAlY transition layer is greater than 1 MΩ at 1300 K, and that of the thermal oxidation layer and the sputtering alumina composite insulation layer on the FeCrAlY transition layer is about 100 KΩ at 1300 K. Thus, it can be seen that selecting NiCoCrAlY as a transition layer is more conducive to high-temperature insulation.

Wrbanek et al. [87] from the Glenn Research Center of NASA began to study composite insulation layers in 2002. Compared with the traditional single-layer insulation layer, the application of the composite insulation layer is more conducive to eliminating pinholes running through the insulation layer in the vertical direction. In this study, aluminum oxide and stainless steel were used as test substrates, and different insulation layer combinations and deposition processes were used. It was found that the composite insulation layer of 1 μm CrC and 4 μm Al_2_O_3_ deposited by the electron beam on the stainless-steel substrate cleaned with H_2_SO_4_/H_2_O_2_ can obtain good high-temperature insulation: the resistance is 84 MΩ at 690 °C and 20 MΩ at 750 °C. The composite insulating layer of 1 μm ZrO_2_/Y_2_O_3_ and 4 μmAl_2_O_3_ deposited by the electron beam on the alumina matrix, polished and cleaned with silica, can also obtain good high-temperature insulation: 50 MΩ at 690 °C, 17 MΩ at 750 °C, and 1.8 MΩ at 900 °C. Table 4 summarizes the high temperature characteristics of different insulating materials.

(3) Coil deposition process

There are generally two methods for coil deposition: PVD (physical vapor deposition) and chemical vapor deposition (CVD). The PVD technologies include evaporation deposition and sputtering deposition. The principle of sputtering coating is that electrons collide with argon atoms in the process of accelerating to the substrate under the action of the electric field, ionizing a large number of argon ions and electrons, and the electrons fly to the substrate. Vacuum evaporation is the vacuum chamber with a substrate pumped into a vacuum, and then the evaporation of the plating material is heated, so that its atoms or molecules from the surface of the gasification escape, the vapor flow forms, there is incident on the substrate’s surface, and the condensation forms a solid film technology. The vacuum evaporation method is advantageous because its equipment is simple, it can save on raw metal materials, has uniform surface adhesion, has a short production cycle, is environmentally friendly, and has the potential for large-scale production, among other advantages; however, given its disadvantages, such as having a higher reaction temperature, its precision control is unfavorable for the thin film deposition process. Compared with the magnetron sputtering process, the electroplating process has the advantages of a fast deposition rate, strong covering ability, strong leveling ability, and equipment economy, but it also has disadvantages such as the difficulty of controlling the composition of the coating and poor process stability. Compared with the magnetron sputtering process, the electroplating process has the advantages of a fast deposition rate, strong covering ability, strong leveling ability, and equipment economy, but it also has disadvantages such as the difficulty of controlling the composition of the coating and poor process stability. Vereecken et al. have successively studied the interaction mechanism between accelerators and chloride ions [52,53,54,55], and Pierre and Gabrielli of Marie Curie University studied the mechanism of copper plating [88]. Chemical vapor deposition (CVD) is a thin film deposition technique in which reactants (usually gases) are formed into solid products in a reaction chamber and deposited on the surface of a wafer by chemical reaction. As the reactant molecules in CVD can adsorb or diffuse on the surface of the substrate many times before decomposition and chemical reaction to form a film, which allows the reactant molecules to reach anywhere on the surface of the substrate, CVD can provide better step coverage than the physical CVD. The following two tables show the comparison of different coil deposition processes and copper films prepared by different deposition processes. Table 5 and Table 6 compares different coil deposition methods.

### 3.3. Printing Process

The printing of electrons is carried out to transform specific functional materials into liquid ink (ink, paste). According to the design requirements for electronic devices and the products’ performance, the film, which is large in scale and flexible, has the same volume as the electronic components (in whole or in part due to the printing or coating technology), and the system is characterized by a by-product production process. In terms of the manufacturing process, the electron-printing process is a part of additive manufacturing technology. Compared with the traditional integrated circuit process, it does not need complex processes, can reduce the loss of raw materials, and is conducive to environmental protection. The prepared products have the following characteristics: being large in area, lightweight, flexible, and bendy.

#### 3.3.1. Micro-Contact Printing Process

Soft etching technology is based on elastic seals with a micro/nanostructure to prepare high-resolution patterns, combining the advantages of nanomaterial technology, fine processing technology, contact printing technology, interface science, and many other technologies, and has attracted wide attention in the field of materials science. This technology was proposed by the American scientist, Whitesides [89], in the late 1990s, and mainly combines top-down lithography technology and bottom-up self-assembly technology. Microcontact printing is the most widely used soft etching patterning technology, and has attracted much attention due to its fine size (micro/nano) and simple patterning process. The elastic seal material used is polydimethylsiloxane, which can be printed on rough surfaces or other surfaces, and the highest resolution is below 100 nm.

**Figure 9:**
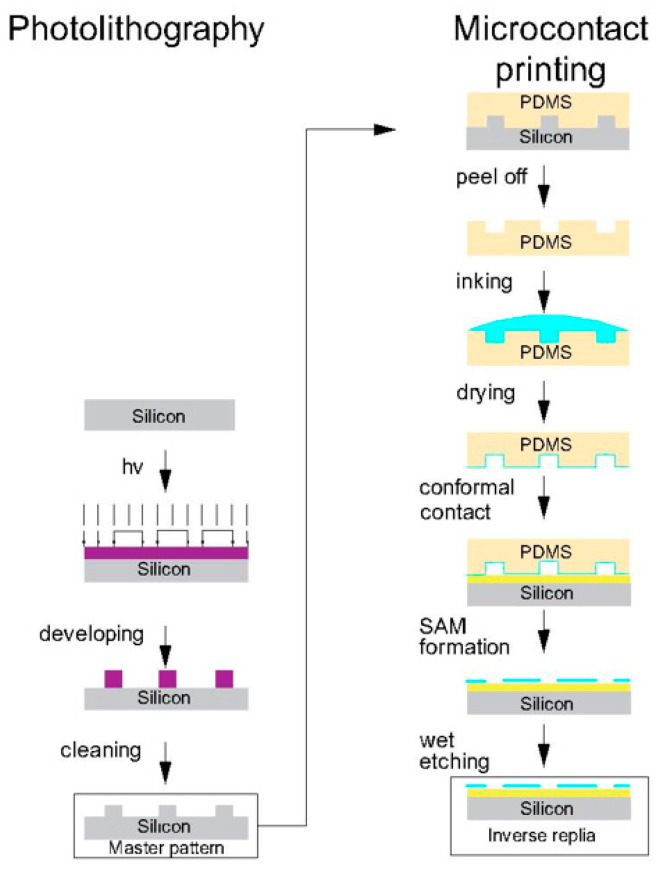
Flow chart of micro-contact printing.

Micro-contact printing is a type of contact printing, and mainly includes elastic seal preparation, contact printing, pattern formation, and other processes. The details are as follows: polydimethylsiloxane (PDMS) is first cast on the silicon template with a microstructure, and then cured and exfoliated to obtain the replica PDMS elastomeric stamp. The “ink” (usually mercaptan alkanethiols) is then dipped and transferred to an Au-coated substrate by contact to obtain a self-assembled monolayer, which can be used as a template or the layer that cannot be easily etched to further prepare high-precision nanostructure patterns. A flowchart is shown in Figure 9 [90].

Printed substrate materials are not limited to gold; other metals are also successfully used as substrates to achieve pattern replication, such as Ag [91], Cu [92], and Pd [93]. The low cost and simplicity of the technology have inspired interest in creating smaller patterns with higher edge resolution and a broader versatility of the technology.

The seal deformation during the removal of the seal from the template, and the contact with the substrate, limits the resolution of the pattern. The PDMS crosslinking process usually leaves some fragments, which have a low molecular weight, uncured, which may pollute the substrate during contact, and thereby reduce the printing quality. When ink molecules contain polarity, the transfer of these impurities enhances the expansion of impressions in almost all organic solvents, thereby changing the size and shape of the protrusions. In order to improve the stability of the seal and the applicability of the printing process, some new seals were invented. Schmid et al. [94] developed a hard PDMS (h–PDMS) that is more suitable for submicron graphics transmission. There are other new seal materials to achieve better mechanical properties. For example, poly(styrene–b–butadiene–styrene) and poly(styrene–b–(ethylene–co–butadiene)–b–styrene) [95] have a high modulus and toughness compared with traditional PDMS. Compared with PDMS stamps, poly(ether ester) [96] was used as a stamp material to accurately map proteins on the surface with a lower ink concentration and time. Lee et al. [97] developed a UV-curable impression material based on a functional prepolymer, which has acrylate groups for crosslinking and different monomer modifiers, and was successfully used to print sub-100 nm hexathiol patterns on gold. The mechanical properties of poly (polyurethane acrylate) impression can be adjusted by changing the modulator.

**Figure 10:**
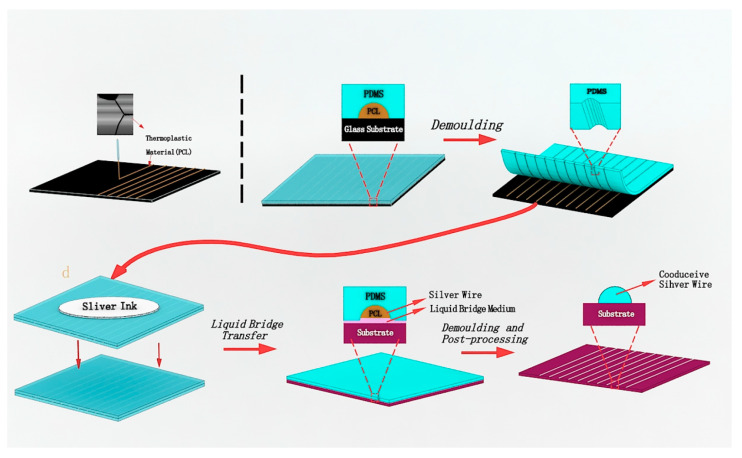
The schematic diagram for the fabrication of the TCE.

Microcontact printing can be patterned at micron and sub-micron scales of various materials, and can achieve small molecules, polymers, and the transfer printing of other materials. It is widely used in biology, materials, and microelectronics. In the field of microelectronics, Xu [98] et al. achieved a flexible transparent conductive electrode with a large aspect ratio and a high-resolution metal mesh transparent electrode with high efficiency and low cost using micro-contact nano-imprint printing. That is, the researchers used an electric field-driven direct writing technology for molten deposition and a liquid bridge micro-transfer printing composite process. It has obvious advantages in terms of its low cost and potential for mass manufacturing, and provides a new solution with prospects for industrial applications in transparent electrode manufacturing. Figure 10 depicts the flow chart of the process.

Figure 10 shows the first process of fabricating the PCL (Polycaprolactone) master mold using the electric-field-driven fusion direct printing technique. Secondly, vacuum casting, or scraping the liquid PDMS on the surface of the PCL master mold and curing it, occurred. Thirdly, peeling off the cured PDMS working soft mold from the PCL master mold occurred. Next, filling and preliminarily curing the nano-silver ink into the grooves of the microstructure of the PDMS soft mold occurred, followed by transferring the silver wire to the target substrate using the liquid bridge transfer process. Finally, peeling off the PDMS soft mold and sintering the transferred silver wire to form the TCE occurred (Transparent Conducting Electrodes).

#### 3.3.2. Transfer Printing Process

The term ‘flexible electronics’ refers to the micro/nano electronic devices made on a flexible substrate. Flexible electronics are flexible, malleable, and biocompatible, and have been widely used in medical diagnosis, information detection, energy collection, and other fields. They have become the frontier of international research. In photolithography, soft etching, nano printing, and other processes, there are high-temperature, physical or chemical corrosion processes, but in this environment, the performance of flexible polymer substrates will be significantly reduced. In order to solve the above problems, transfer printing technology is used in the production of extensible, flexible electronics. Transfer printing technology is compatible with the semiconductor and nano manufacturing process and can highly adapt to various planar and non-planar structures. The basic principle of transfer printing is to use the difference between the two substrates and functional devices so that functional devices transfer from one substrate to another substrate, which can solve the problem of inorganic conductive materials not being able to directly grow and process on a flexible substrate. The existing transfer printing methods mainly include the rate-based transfer printing method [99,100], the micro-structure-based transfer printing method [101], the tape-based transfer printing method [102,103], and the sacrificial layer transfer printing method [104,105], according to their published time arrangement. Table 7 is the commonly used transfer printing method.

Zhigang Wu’s research group [106] proposed the “one-step transfer printing method” for the manufacture of flexible stretchable electronics by using the rate-based transfer printing method. Graphics of different widths were made on the donor substrate, then the copper structure was transferred using pre-stretched PDMS, resulting in a structure that could still be used when it is stretched by 300%. Kim’s research group [101] designed a PDMS seal into a micro pin-like structure. By applying different pressures on donor and recipient substrates to control the transfer printing process, the silicon structure was transferred to the quartz surface, and the researchers studied the mechanism via which this structure regulates transfer printing with different pressures. The transistor is fabricated and tested by this transfer printing method. Ko’s group [107], from the Ulsan National Science and Technology Institute, studied the process of the absorption and release of octopus tentacles, and found that changes in humidity can regulate the change in viscosity. From this, the researchers were inspired, and developed the PDMS surface using the stamp of the micro-hole array.

The tape transfer printing method [103] uses tape as a seal for transfer printing. Many tapes have special properties, such as water-soluble tape that can dissolve in water and heat-release tape which loses viscosity after heating. Lin Yuan’s research group [108] from the University of Electronic Science and Technology of China used the heat-release tape as the seal. When the temperature of the tape was lower than the release temperature, the functional structure with gold and PI could be transferred to the stamp. Then, the tape with a functional structure was placed on the PDMS recipient substrate for heating, and the tape lost viscosity and fell off. Xu’s team at the University of Illinois [100] used water-soluble tape to transfer the active material of a lithium-ion battery, printed it on a recipient substrate with electrodes, and finally, dissolved the tape with water to produce a flexible and stretchable lithium-ion battery array. Figure 9 shows the tape transfer method.

With the development of micro/nano processing technology, the scope of application is becoming increasingly extensive, and the size of transfer printing graphics is developing towards miniaturization and high integration.

In terms of miniaturization, as can be seen from Table 7, the resolution of the functional structure transferred by most transfer printing methods is still only at micron or submicron scales. Although some methods conduct transfer printing at the nanoscale, the materials used for transfer printing are only suitable for metals. Based on this problem, Pang [109] and others, for the first time, put forward a new kind of auxiliary nanoscale transfer printing method with a sacrificial layer. This paper expounds on the sacrificial layer transfer process to improve the resolution and reduce the cause of the dependent variable. This method is applied to the manufacture of flexible electronics, flexible LED circuits, and flexible capacitors and tests, which demonstrates the good performance and high versatility of this method.

In terms of integration, Nishana [110] et al. discussed a dense array seal of active elastic composite material, which adopts an expandable 4 × 4 geometric design and a multi-way interconnection scheme to ensure a small footprint. It consists of 16 individual seals, each with a lead zirconate titanate (PZT) actuator and a strain gauge sensor. The drive and sensing characteristics and closed-loop feedback control were established, and the performance of the dense array seal was verified using selective pick-and-place micro-transfer printing experiments, which greatly improved efficiency and productivity.

Through the continuous integration of micro and nano processing technology in transfer printing technology, the transfer printing method is more and more extensive; however, transfer printing technology is in urgent need of a new method that can enable the resolution of the functional structure of transfer printing to reach the nanometer level, but it also needs to be highly integrated to improve the efficiency of transfer printing. Moreover, the method is as simple as possible, and is not affected by the nature of the donor substrate, the stamp, and the recipient substrate. To achieve such a breakthrough, transfer printing technology will progress greatly.

#### 3.3.3. Ink-Jet Printing Process

Ink-jet printing technology is a complex and diverse new technology. It applies control system outputs, can print a single or diverse power supply circuit, and can support a variety of design schemes and data types, with a strong compatibility mode. Based on the control of the computer and printer, the nozzle is sprayed with a certain amount and a certain frequency of ink on the surface of the matrix, and high-precision and high-quality pattern information is drawn on the matrix, and the circuit design and electrode fabrication are completed. As the nozzle aperture is generally less than or equal to 5 μm, the extrusion of micro ink droplets spread on the substrate-formed ink dot area is small, so the printing line has good line accuracy. Ink-jet printing technology can replace the traditional printing process by using organic materials and metal composite materials based on the aqueous solution method. Ink-jet printing can be divided into continuous printing and on-demand printing according to its working principle.

Traditional ink-jet printing is the most common method of coating nano-silver ink; however, it is often necessary to adjust the preparation of high-quality nano-silver ink to match, although this also increases the development and manufacturing costs of nano-silver ink. The airflow jet printer and electrohydrodynamic ink-jet printer are introduced below.

**Figure 11:**
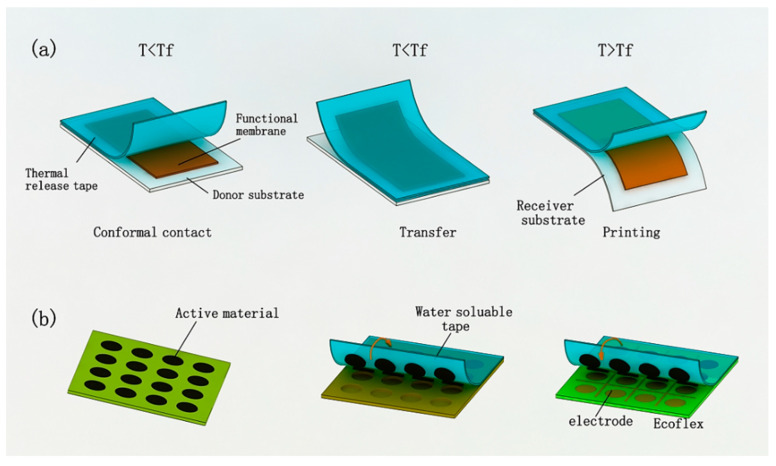
Tape-assisted transfer: (**a**) heat-release tape transfer, (**b**) water-soluble tape transfer.

**Figure 12:**
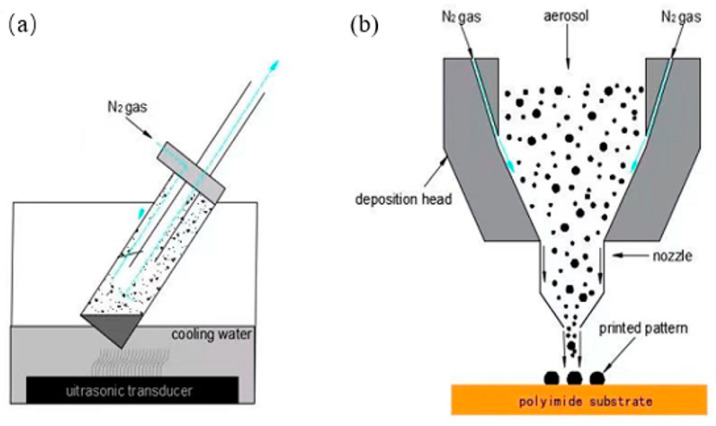
Schematic diagram of the working principle of the jet printer: (**a**) atomization of ink, (**b**) inkjet process.

Jet printing is a coating method that requires the lowest properties of nano-silver ink. Figure 11 shows the working principle of the jet printer [111]. In Figure 12a, the nano-silver ink is atomized first, and the ink is dispersed into liquid particles and mixed with working gas to form aerosols. In the process of air-jet printing (Figure 12b), the size of the atomized ink is about 1–5 μm, and these droplets are sent to the nozzle by high-speed air. The nozzle part will produce a ring around the airflow, aerodynamically focusing on the droplets ejected, so that the aerosol ink ejects into a stable fine line to ensure that the aerosol ink point is controlled within less than one-tenth of the diameter of the nozzle. The thinnest nozzle can print a line with a width of 5 μm. At the same time, because the ink beam at 2–5 mm of the height of the width is the same, some of the substrates can maintain the same thickness of the print line.

**Figure 13:**
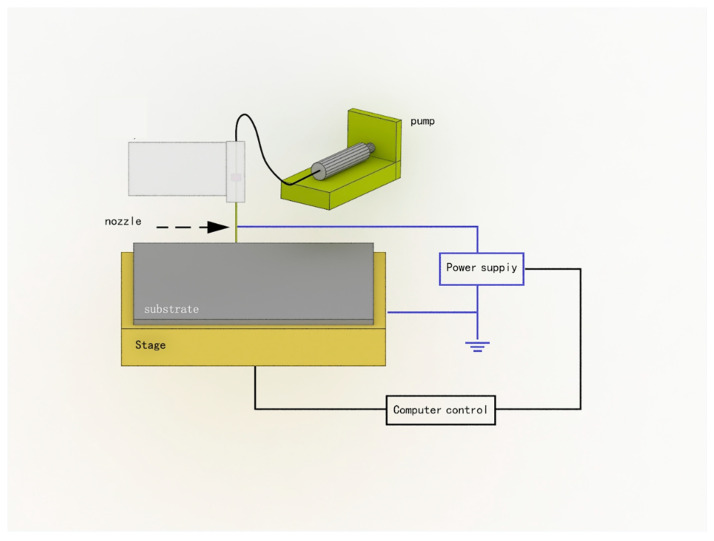
Ink-jet printing schematic diagram of the electric fluid power.

Mahajan A et al. [112] prepared a complete nano-silver coating on a fluted substrate using this method, with a line width and spacing of about 50 μm. Electrohydrodynamic ink-jet printing uses high-voltage, electric field-stretching ink to pull the jet from the top of the meniscus and deposit it on the substrate to form a coating, as shown in Figure 11 [113]. The nano-silver ink was rheologically induced to form a Taylor cone under the action of a high-pressure electric field, and then the micro–nano jet with a diameter much smaller than the inner diameter of the jet hole was formed. The micro–nano coating was deposited on the substrate. Compared with the traditional ink-jet printing method, the resolution of the electro-fluid printing method is very high, at less than 1 μm; an applicable ink viscosity range of 1–10,000 CP is also possible. Electrohydrodynamic ink-jet printing has attracted increasing attention due to its wide range of applicable ink properties and high printing accuracy. Electrohydrodynamic ink-jet printing is used to print nano-silver ink, and the coating with a line width of 50 nm and spacing of about 500 nm can be printed at the finest level. Figure 13 shows the principle of electro-hydraulic power in inkjet printing.

#### 3.3.4. Introduction to Conductive Ink Printing

As the core material of printed electronic technology, conductive ink is one of the keys to the development of printed electronic technology. At present, the main components of conductive materials in conductive ink include carbon materials, conductive polymer materials, nano metal materials, and many kinds of mixed conductive materials. Nano metal materials have become a popular conductive component because of their excellent electrical properties and are made into conductive ink. Nano metal materials, which are commonly used as conductive components in conductive ink, include nano-gold, nano-silver, and nano-copper. Among the various conductive inks, silver- and carbon-based inks are preferred, whereas copper-based inks are the most profitable. Copper, however, has problems with oxidation, making it less suitable for printing. Nanoparticle- and particle-free alternatives are also springing up, but they cost more. Nanometer silver materials have great research significance and application prospects because of their excellent electrical conductivity, stable oxidation resistance, and relatively low manufacturing cost. This article introduces several conductive ink products already sold in the market. Table 8 is an introduction to some products.

For all the products listed above, CON–INK550 is a new electronic material for digital print-deposited conductive circuit technology. It is compatible with Epson series nozzles (DX5, DX7), Fuji Star series and Spectra series Konica nozzles (KM512i, KM1024i), and Ricoh nozzles (Gen5, Gen5s, Gen6). Its advantages include its excellent electrical conductivity, extremely smooth printing, high nano-silver content, and good hardness and adhesion. EI–1104 and EI–906 are two products from Electroinks, a leader in particle-free conductive metal inks and advanced materials. EI–1104 inks are formulated for ink-jet deposition and applications requiring high electrical conductivity. This unique ink provides stable injection performance at room temperature and has a long shelf life. The low curing temperature makes the EI–1100 series suitable for PI, glass, and silicon nitride. The conductive material of EI–906 is composed of silver and silver chloride, and its viscosity is very high, up to 16,000 cps, so it is suitable for screen printing.

In recent years, research on nano–metal–conductive ink for ink-jet printing has mainly been focused on improving the solvent formula properties of conductive ink to optimize the molding process and coating quality of spray printing coating or to optimize the electrical and mechanical properties of the coating under different molding conditions. Liu et al. [114] from the Harbin Institute of Technology proposed a method to prepare nano-silver ink by compounding the nano-silver particles of different sizes. The principle that large, slow-moving silver particles hinder small silver particles from moving toward the edge of the droplet during the drying process of ink droplets was adopted to avoid the coffee ring phenomenon of printing coating. The microstructure density, electrical conductivity, and bending reliability of the silver coating were improved. Teng et al. [115] dissolved silver neodecanoate in xylene to form silver conductive ink. The ink was transferred to the surface of solar cells by a direct ink-jet method, and the silver conductive mesh was obtained by a heat treatment at 350 °C. Valeton et al. [116] used the ink-jet printing method to deposit organic silver-based ink. After UV curing and hydroquinone solution treatment, a silver conductive layer was formed. Cai et al. [117] dissolved silver oxide in the methanol solution of ammonium carbamate to obtain particle-free conductive ink. Using laser direct writing technology, the organic silver compound printed on polyimide film was decomposed into silver particles, and finally the silver film with good conductivity was obtained.

With the increasing popularity of various flexible substrates in the microelectronics industry, conductive ink for printing electrons is developing in the direction of low cost, high conductivity and a low-heat treatment temperature. Particle-free conductive ink has become the focus of future development.

### 3.4. Other Processing Methods

#### 3.4.1. Liquid Metals

**Figure 14:**
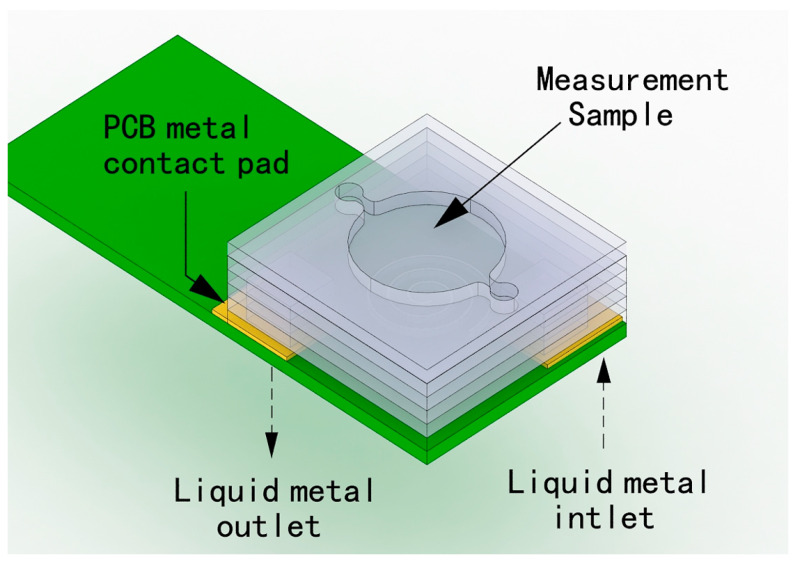
Liquid metal preparation of miniature coils.

The key advantage of using liquid metal technology is that it allows flexible, complex 3D electrical conductors to be fabricated within microchannels with very few fabrication steps, compared with using conventional machining methods to fabricate 3D micro coils [118]. The liquid metal structure has the ability to deform mechanically and can be applied to many devices that require high precision, high complexity, and high mechanical strength, such as tunable fluid antennas and pressure sensors [119]. The method of fabricating a multi-layer integrated micro coil using liquid metal is shown in Figure 14. First, the structural layer is processed by laser layering. The micro coil’s structural layer is composed of three layers: a spiral channel, an interconnection, and a lead-out channel. All layers are assembled together via a stacking process. An adhesive glues the location of the wire inlet and outlet of the coil to the top of the PCB metal pad. Firm and stable contact between the micro coil structure’s layer and the liquid metal is achieved through contact with the PCB. Through-holes with a diameter of 1 mm are mechanically drilled in the contact plate of the PCB. Next, liquid metal is injected from the back of the PCB, allowing the liquid metal to flow through the first copper pad on the PCB and into the spiral channel. The liquid metal flows through the interconnected layer, then through the lead-out channel back to another PCB contact pad, and finally out through the outlet drilled in the PCB. The large contact area between the liquid metal and the PCB contact pad ensures a good electrical connection with the external equipment, and the liquid metal is cooled to form a three-dimensional coil [120], which completes the preparation process of the liquid metal micro coil.

#### 3.4.2. Femtosecond Laser

With the continuous improvement of the integration and complexity of microsystems, the requirements for the preparation of three-dimensional spiral micro coils continue to increase, and the performance of micro coils as the basic components and important functional parts of microsystem chips directly affects the overall performance of microsystems. Moreover, the preparation of high-performance micro coils has a very important practical significance [121,122,123]. The use of femtosecond laser micromachining technology can be prepared more uniformly inside the transparent medium, has good performance, and is able to meet the actual application requirements of microsensor devices. Moreover, the preparation of three-dimensional spiral micro coils is currently a relatively advanced technology, and is based on three-dimensional micromachining femtosecond laser technology to prepare three-dimensional high-performance coils, which is mainly combined with the idea of metal “micro-curing”, as proposed by Siegel et al. in 2007 [124]. The process of preparation using femtosecond laser micromachining technology is mainly divided into three steps. First of all, the femtosecond laser modification process is used to scan the spiral microchannel structure inside the material. More specifically, a femtosecond laser is used to focus on the substrate through the microscope, and laser scanning modification is carried out through a 3D processing platform and well–written program. Then, the sample undergoes wet etching, that is, the modified sample is placed in the corrosion liquid, with ultrasonic oscillation at room temperature. The purpose is to make the modified part of the sample fully react with the corrosive liquid to obtain the internal channel of the material. After the preparation of the above two steps, the internal hollowed-out microchannel is prepared. Finally, the metal is micro-cured, the sample is tightly bonded to the PDMS film, the needles of the two syringes are inserted into the PDMS, and a micro-injection pump is connected to each of the other two ends of the two entry syringes of the micro coil to heat and melt the gallium metal and inject it into a syringe. Pushing the gallium metal syringe inward while the other syringe inhales outward, the gallium metal fills the entire micro coil channel. The ultrasound should then be cooled to room temperature to peel off the PDMS film to obtain the prepared micro coils.

This method of preparing a three-dimensional spiral micro coil using femtosecond laser modification, wet etching, and metal curing can prepare a micro coil with any morphology, and during the preparation process, the parameters of the micro coil can be precisely controlled, and the accurate processing of the three-dimensional channel can be realized. By writing a program to control the 3D platform to move along the designed trajectory, it is easy to achieve the processing of any shape of 3D microchannels. Compared with the use of the planar process after multi-layer mask preparation and layer-by-layer mask accumulation to prepare three-dimensional structural coils, this preparation method is complex and expensive. Moreover, by winding metal wires on the surface of the cylinder or plating spiral conductive wires on the surface of the cylinder, the femtosecond laser wet etching technology can prepare a micro coil that can be integrated inside the material, and there is no such complex process of layer accumulation, while more accurately making any shape of micro coil, which ensures the consistency and superiority of the performance of the micro coil. In particular, it can reduce dispersion and anti-interference, and can also flexibly increase or decrease the number of turns as needed, with a high degree of integration, which is convenient for integration with other micro coils and even microsystems. Both these micro coils and femtosecond laser wet etching techniques will play an important role in microcircuit systems.

## 4. Challenges and Prospects

Among the various micro metal coils summarized in this paper, the manufacturing cost of macromachining is low, but there are many processing steps. Additionally, the manufacturing resolution is low, and the RF field is not uniform. MEMS processing can make the coil size as small as possible, can increase the performance of the device, and can achieve mass production to reduce the cost. Additionally, it can be integrated with machinery, materials, manufacturing, information and automatic control, physics, chemistry, biology, and other disciplines. It has a wide range of applications in, and lays a good foundation for, the Internet of Things era, which is characterized by the “perception of everything, full of wisdom” principle; however, MEMS processing technology still faces many new problems, such as how to protect the microstructure during silicon wafer cutting to prevent silicon dust from damaging the chip. Other issues include how to prevent adhesion between moving parts and substrates in the process of releasing the microstructure, as well as stress release in device packaging and the standardization of packaging and interfaces. In addition, the reliability and reliability evaluation of packaging performance are also discussed.

As a kind of electronic manufacturing technology, the electron printing process has advantages that a silicon-based integrated circuit does not have, including its large area, flexibility and low cost, and it plays a pivotal role in MEMS and the manufacturing of other micro coils. Micro contact printing can realize the transfer printing of small molecules, polymers and other materials, and is widely used in biology, materials, and microelectronics; however, the resolution and transfer efficiency of the transfer function structure are low. Liquid metal processing makes the metal coil have a good mechanical deformation ability, which is suitable for devices requiring high precision, high complexity and high mechanical strength processing. Femtosecond laser micromachining technology is more practical in preparing 3D spiral micro coils with better performance. Table 9 shows the performance and practical applications of coils prepared based on different processes.

In engineering practice, higher requirements are put forward for the development of micro metal coils, and there are some technical difficulties in the preparation process. In order to promote the further development of the Internet of Things’ (IoTs) industry, there are still some challenges in preparing high-performance, low-cost, mass-produced miniature metal coils, and the following future trends are worth paying attention to:

(1) The area of single-layer planar metal coils inevitably increases with the increase in coil turns, but the preparation process of multi-layer coils is more complicated than that of single-layer coils. In addition, due to the superposition of insulating layers, it is difficult to achieve surface planarization, which will greatly affect the photolithography effect. The effect is not improved significantly due to the limited number of accumulated layers; therefore, it is necessary to study a new type of multilayer coil with a simple and reliable preparation process and high-quality factor.
(2) Rigid substrates cannot be applied to the measurement of some non-planar surfaces and flexible surfaces. In flexible substrate processing, some links need a high-temperature environment, which will cause damage to the flexible film, in order to improve the magnetic properties. Thus, there is a need to develop more suitable methods for the combination of the materials and the substrate.
(3) The coil size is small, the degree of integration is high, and the working temperature is increased accordingly. For the vast majority of insulating materials, their resistance will decrease exponentially with the increase in the temperature at high temperatures, and the insulation performance will decrease greatly; therefore, it is necessary to study the preparation of the insulating layer of composite insulating materials with higher temperature resistance and stability.
(4) For the electronic printing process, the resolution of the functional structure transferred by most transfer printing methods is still only at a micron or sub-micron scale, so it is necessary to study a new transfer printing technology, which can improve the resolution of the functional structure of transfer printing and transfer efficiency.

## Figures and Tables

**Table 1:** A summary of some examples of typical processing methods.

Coil Type	Ref.	Size	Coil Materials	Applications
Processing on PCB	Wei et al. [68]	5 mm	copper	NMR probe
	Tang et al. [56]	4.6 mm	copper	Isolated Switching Power Converters
Hand winding	Peck et al. [69]	50 μm–1.8 mm	copper	NMR detection in the laboratory
Wire bonding	Kratt et al. [70]	300 μm	insulated bondable gold wire	Experiment

**Table 2:** Metal coils with different structures based on MEMS processing technology.

Coil Type	Ref.	Special Design Structure	Size	Resistance (mΩ)	Frequency (MHz)	Inductance Value (nH)	Q
Single-layer planar coil	Yamaguchi M, et al. [46]	Sandwich construction	–	6800	1000	7.7	7
					2000	7.9 × 10^6^	12.7
	Peng S, et al. [75]	Sandwich structure with electroplated NiFe as the magnetic core	1.7 × 1.7 mm^2^	470	21.7	204	–
					9.2	–	9.23
	Olivo J, et al. [77]	High thickness spiral inductor, coil thickness is 60 um	14.88 × 2 mm^2^	1058	5	460	13.65
Multilayer planar coil	Xuming Sun, et al. [51]	Parylene-based 3D high-performance folded multilayer inductors	2.1 × 2.1 mm^2^	–	4.1	427.9 (A layer of the coil)	7.48
						12,790 (Six layers of the coil)	10.68
Solenoid coil	Dragan Dinulovic, et al. [48]	Soft magnetic CZT (Co–Zr–Ta) material as the core material	2.6 × 2.4 × 0.4 mm^3^ (L × W × T)	320	15	100	–
					30	97	15
	Lei Gu, et al. [83]	The solenoid inductor is embedded in the silicon cavity	–	1270	5350	45	–

**Table 3:** Summary of coil applications with different substrate materials.

Type of Substrate	References	Substrate Materials	Coil Materials	Advantages	Applications
rigid	Jiang Q, et al. [27]	Glass	Cu	High light transmittance, high hardness, corrosion resistance	ME sensor
	Wang N, et al. [63]	Si	Cu	Excellent piezoresistive properties, easily oxidized to form a layer of silica on the surface (insulating layer)	Micro-transformers
flexible	Xuming Sun, et al. [51]	Parylene substrate	Cu	Biocompatibility, flexibility, chemical inertness and optical transparency	Wireless power transmission applications
	Woytasik M, et al. [84]	Kapton and Peek	Cu	Physicochemical stability, flexibility	Non-destructive testing (NDT) techniques

**Table 4:** High-temperature characteristics of different insulating materials for metal coils.

Reference	Insulating Layer	Temperature (°C)	Insulation Resistance (MΩ)
Nakai H, et al. [85]	6.5 μm alumina	820	>10
J.Y. Park, et al. [86]	Thermal oxide layer + sputtering alumina on the NiCoCrAlY transition layer	1027	>1
	Thermal oxide layer + the sputtering alumina composite insulation layer on the FeCrAlY transition layer	1027	>0.1
John D, et al. [87]	Composited with 1 μm CrC and 4 μm Al_2_O_3_	690	84
		750	20
	Composited with 1 μm ZrO_2_/Y_2_O_3_ and 4 μm Al_2_O_3_	690	50
		750	17
		900	1.8

**Table 5:** Features of different processes for metal deposition.

Process	Deposition Rate	Sedimentary Area	Cost of Equipment	Equipment Complexity	Difficulty Level
Electroplate	Quick	Large	Low	Low	Simple
Sputtering deposition	Medium	Large	High	High	Medium
Vacuum evaporation	Slow	larger	High	Medium	Medium

**Table 6:** Comparison of copper film growth in various methods.

Methods	CVD	PVD	Electroplating	
			Electroless	Electrolytic
Impurities	C, O	Ar	Seed layer	–
Deposition rate (nm/min)	~100	≥100	<100	~200
Process temperature (°C)	~250	RT	50~60	RT
Step coverage	Good	Fair	Good	Good
Via fill capability	Good	Poor	Fair–poor	Fair–poor
Environmental(waste)	Good	Good	Poor	Poor

**Table 7:** Commonly used transfer printing methods.

Methods	Transfer of Material	Stamp	Acceptor Substrate	Sizes (μm)
Rate-based transfer printing method	Si	PDMS	GaAs, InP	0.3
	Cu	PDMS	PDMS	100
	PZT	PDMS	PDMS	300
	Ag/MgF2	PDMS	PDMS	0.225
	GaAg	PDMS	PI	–
Micro-structure-based transfer printing method	Si	PDMS	Quartz	100
	InGaAs	PDMS	PI, PET, Si	50
	Au, Ag, Cu	Hydrogels	PET, PVC	20
	Si	PDMS	Glass	15
Tape-based transfer printing method	LiCoO_2_/Li_4_Ti_5_O_12_	Tape	Ecoflex	1580
	Si	Tape	PI	250
	AuNPs	Tape	Al	10
	Au/PI	Tape	PDMS	–
Sacrificial layer transfer printing method	MOS device	PDMS	PDMS	–
	α−Si	PDMS	PET	–
	SWNT	PU	PI	–

**Table 8:** Conductive ink products.

Product	Manufacturer	Conductive Material	Solid Content	Viscosity	Applicable Process
EI–1104	Eletroinks	Ag	14 wt%	10 cps	Ink-jet printing
EI–906	Eletroinks	Ag/AgCl	30 wt%	16,000 cps	Silk screen printing
Ink10	FUDY	Ag (10 nm)	35 wt%	5–30 cps	Ink-jet printing
CON–INK550	Dahua Brocade	Ag (30 nm)	25–30 wt%	5–6 cps	Ink-jet printing

**Table 9:** The performance and practical applications of coils prepared based on different processes.

Processing Technology	Device	Advantage	Application
Macro processing method	Detection probe	Low cost, not easily oxidized	Detection equipment such as NMR machines
	Manual winding coil on capillary	The method is simple and suitable for manual operation	Detection of coil preparation parameters under laboratory conditions
	Experimental detection coil	Novel methods with the potential for mass production	Integrated micro-inductor
MEMS Machining Method	WPT system	Wireless transmission, high Q value and high inductance increase the transmission distance and energy	Power supply for devices such as cochlear implants in biomedical fields
	Isolation miniature transformer	Small footprint, high coupling, high-voltage gain and low DCR, suitable for signal conversion in the frequency range of tens of MHz	Automotive electronics, industrial electronics, etc.
	Three-axis magnetic sensor	Surface sensor detection	Wearable equipment, nondestructive testing (NDT)
	Energy harvesting	Large functional density and small size of the devices	Health and Use Monitoring System for Defense Military Helicopters (HUMS)
Printing technology	Array electromagnetic Sensor	High density, microwire width detection coil	Nondestructive electromagnetic testing field
	Transparent conductive grid	Large aspect ratio, high resolution, large area fabrication	Touch screen, organic solar cell, transparent display
Liquid metals	Multilayer integrated NMR microcoil	Good mechanical deformation ability	Tuned fluid antenna, pressure sensor, energy harvester and other devices need high precision, high complexity, high mechanical strength processing
Femtosecond laser	3D spiral microcoil	More uniform, good performance, able to meet the practical application requirements	Micromechanical systems, microelectronic devices, micro sensors and other fields of micro-system integration

## Abbreviations

MEMS	Micro-Electro-Mechanical System
RF	Radio Frequency
PI	Polyimide
PDMS	Polydimethylsiloxane
PVD	Physical Vapor Deposition
CVD	Chemical Vapor Deposition
CMOS	Complementary Metal Oxide Semiconductor
SECT	Silicon-Embedded Coreless Transformer
DLM	Double-Layer Metal
PCB	Printed Circuit Board
NMR	Nuclear Magnetic Resonance
UV	Ultraviolet
PCL	Polycaprolactone
